# Expression Profile of CYP1A1 and CYP1B1 Enzymes in Colon and Bladder Tumors

**DOI:** 10.1371/journal.pone.0082487

**Published:** 2013-12-16

**Authors:** Vasilis P. Androutsopoulos, Ioannis Spyrou, Achilles Ploumidis, Alexandros Eystathios Papalampros, Michalis Kyriakakis, Demetrios Delakas, Demetrios A. Spandidos, Aristidis M. Tsatsakis

**Affiliations:** 1 Laboratory of Toxicology, Department of Morphology, Faculty of Medicine, University of Crete, Heraklion, Crete, Greece; 2 First Department of Surgery, University of Athens, Laiko Hospital, Athens, Greece; 3 Department of Urology, “Asklipeio” General Hospital, Voula, Athens, Greece; 4 Laboratory of Clinical Virology, Department of Laboratory Medicine, Faculty of Medicine, University of Crete, Heraklion, Crete, Greece; Univ of Bradford, United Kingdom

## Abstract

**Background:**

The cytochrome P450 CYP1A1 and CYP1B1 enzymes are involved in carcinogenesis via activation of pro-carcinogenic compounds to carcinogenic metabolites. CYP1A1 and CYP1B1 have shown elevated levels in human tumors as determined by qRT-PCR and immunohistochemical studies. However studies that have examined CYP1 expression by enzyme activity assays are limited.

**Results:**

In the current study the expression of CYP1A1 and CYP1B1 was investigated in a panel of human tumors of bladder and colorectal origin by qRT-PCR and enzyme activity assays. The results demonstrated that 35% (7/20) of bladder tumors and 35% (7/20) of colon tumors overexpressed active CYP1 enzymes. CYP1B1 mRNA was overexpressed in 65% and 60% of bladder and colon tumors respectively, whereas CYP1A1 was overexpressed in 65% and 80% of bladder and colon tumors. Mean mRNA levels of CYP1B1 and CYP1A1 along with mean CYP1 activity were higher in bladder and colon tumors compared to normal tissues (p<0.05). Statistical analysis revealed CYP1 expression levels to be independent of TNM status. Moreover, incubation of tumor microsomal protein in 4 bladder and 3 colon samples with a CYP1B1 specific antibody revealed a large reduction (72.5 ± 5.5 % for bladder and 71.8 ± 7.2% for colon) in catalytic activity, indicating that the activity was mainly attributed to CYP1B1 expression.

**Conclusions:**

The study reveals active CYP1 overexpression in human tumors and uncovers the potential use of CYP1 enzymes and mainly CYP1B1 as targets for cancer therapy.

## Introduction

Bladder and colon cancer are two of the most frequently encountered malignancies worldwide. The 5-year survival rate for bladder cancer is 62% and for colon cancer 64% provided that the tumor has not metastasized [1]. In Europe 105,000 cases of bladder cancer are diagnosed every year, whereas approximately 30,000 cases of bladder cancer result in fatalities annually [1,2]. Colon cancer cases present higher frequencies with approximately 300,000 new cases annually and 140,000 morbidities every year [1,2]. Colon and bladder cancers are categorized to *in situ* carcinomas when the tumor is localized above the basement membrane and to invasive carcinomas when the tumor penetrates the transitional epithelium. The most common form of bladder cancer is carcinoma of the transitional epithelium. The treatment for colon and bladder cancer generally consists of surgery and chemotherapy. The chemotherapeutic drugs used for bladder cancer include the alkylating agent cisplatin and the DNA cross-linker mitomycin C [3]. Chemotherapy for colon cancer includes the antimetabolite 5-fluorouracil (5-FU) and the cisplatin analogue oxaliplatin [4]. Chemotherapy using 5-FU and cisplatin often results in unwanted side effects notably bone marrow suppression and nephrotoxicity. 

Cytochrome P450s are a multigene superfamily of enzymes that play major roles in the detoxification, activation and metabolism of several endogenous and exogenous substances [5]. The first family of CYPs consists of three members CYP1A1, CYP1B1 and CYP1A2. CYP1A1 and CYP1B1 are extrahepatic enzymes that catalyze the oxidation of pro-carcinogens to carcinogenic reactive intermediates [6]. As a result the expression of CYP1A1 and CYP1B1 is an important contributor to carcinogenesis. The role of CYP1A1 and CYP1B1 is not limited to the metabolism of drugs and carcinogens. CYP1 enzymes can metabolize endogenous compounds to metabolites that possess potent biological activities. For example CYP1A1 exhibits hydroxylase activity towards arachidonic acid, whereas towards eicosapentaneoic acid it is an epoxygenase [7]. Both of these polyunsaturated fatty acids are metabolized to products that play important roles in the regulation of vascular tone and of renal, pulmonary and cardiac function [7]. Recent evidence also suggests that the arachidonic acid CYP1-mediated derivative 12 (R)-HETE can serve as a potent activator of AhR activity suggesting a possible involvement in inflammatory disease condition of the skin [8]. More importantly CYP1A1 was reported by Rodriguez and Potter to regulate breast cancer cell proliferation and survival via suppression of AMPK signalling, whereas with respect to cancer metastasis CYP1A1 has been shown to be involved in β-catenin signaling [9-11]. As a result constitutive expression of CYP1 enzymes in tumors may not directly influence cancer progression via activation of pro-carcinogens as other important biological pathways are linked to the functional role of these enzymes, irrespective of their metabolic capacity towards xenobiotics.

Differential expression of CYP1A1 and CYP1B1 in various tumor types, compared to normal tissue has been demonstrated by several studies, thus highlighting the potential use of the two CYP1 isoforms in cancer prognosis [12-15]. In addition selective overexpression of CYP1A1 and CYP1B1 may be utilized to target specific tumor types by the activation of non-toxic prodrugs that are selectively metabolized to cytotoxic products [16-18]. No significant progress has been made in targeting CYP1B1, but key papers on CYP1A1 targeting with small molecules have recently been published [19-21]. More importantly extrahepatic expression of CYP1B1 may influence the response of patients to chemotherapy, as some frequently used chemotherapeutic drugs such as tamoxifen, taxol and flutamide are substrates for CYP1B1 [17,22]. Thus CYP1A1 and CYP1B1 play essential roles in cancer therapeutics, as well as carcinogenesis. 

While there is extensive evidence on the expression profile of CYP1A1 and CYP1B1, in terms of mRNA and protein levels in tumors, studies that have examined CYP1-enzyme activity are limited. Since CYP1 enzymes play essential roles in the activation of pro-carcinogens and the metabolism of anticancer drugs and prodrugs it is necessary to substantiate information regarding their activity levels in tumors. The aim of the present study was to examine the expression profile of CYP1A1 and CYP1B1 in a range of human tumors of bladder and colorectal origin. Our observations clearly indicate that CYP1A1 and CYP1B1 are overexpressed in colon and bladder tumors. 

## Materials and Methods

### Chemicals

4´ methoxy 3´,5,7 trihydroxy flavone (diosmetin) was purchased from Extrasyntheze (Genay, France) and 4´,3´,5,7 tetrahydroxy flavone (luteolin) from Sigma Aldrich (Dorset, United Kingdom). Reagents for cell culture were from Sigma Aldrich, whereas solvents for analytical chemistry were from Fisher Scientific (Thessaloniki, Greece). The C18 column for diosmetin and luteolin separation was purchased from Phenomenex (Cheshire, United Kingdom). The cDNA synthesis kit was purchased from Takarra (Osaka, Japan). Polyclonal antibody for human CYP1B1 was purchased from Santa Cruz Biotechnology (Heidelberg, Germany) and polyclonal antibody for CYP1A1 from Millipore (Massachusetts, US). 

### Patients and tumor specimens

Paired tumor and normal tissue samples from a series of 20 patients with primary colon cancer and 20 patients with primary bladder cancer were acquired following routine pathological examination. The clinicopathological characteristics are shown in Table 1. Tumors were categorized into invasive (T2, T3) and non-invasive (Ta, T1). Patients were diagnosed with cancer from medical examination, medical records and biopsy results. The 1973 WHO grading system was used in this study to classify tumor grade of each sample acquired from patients with bladder and colon cancer. 

**Table 1:** Demographic parameters of the subjects used in the study.

Subjects	40
Male	32
Female	8
Age (Mean)	68.7
Age (Range)	36-89
Tumor type (**Bladder**)	20
Tumor type (**Colon**)	20
Stage (**Ta**)	7
Stage (**T1**)	6
Stage (**T2**)	12
Stage (**T3**)	15
Grade (**I**)	4
Grade (**II**)	15
Grade (**III**)	12

Written informed consent forms were obtained for all patients from whom specimens were collected. Each patient acknowledged that tissue will be taken and used in the context of the study and signed the consent form. The study protocol and consent procedure was approved by the Ethics Committee of the University of Crete.

Tissue specimens were collected during surgery from the tumor, placed in 1.5-ml eppendorf tubes and stored at -80 °C for further use. A control sample was collected from the surrounding tissue area for each tumor specimen that was free of neoplasmatic infiltration. 

### RNA extraction and real time PCR

Total RNA was extracted from tumor tissues with Trizol. Briefly tissue fragments were homogenized in 1 ml Trizol, mixed with 200 μl chloroform and centrifuged for 15 min at 13,000 rpm. The top layer of the supernatant was collected and mixed with 500 μl of ice-cold isopropanol and further centrifuged at 13,000 rpm for 10 min. The resulting RNA pellet was washed once with 75% ice-cold EtOH and resuspended in 40 μl of DEPC-treated water. RNA concentration was estimated by spectrophotometry. 

cDNA synthesis was performed using a Takarra RT kit according to the manufacturer’s instructions. Briefly, 1 μg of RNA was mixed with H_2_O and oligo dT primers, heated for 5 min at 25 °C and finally incubated at 43 °C for 1 hr in the presence of DNTPs, reverse trancriptase and reaction buffer containing MgCl_2_ (5 mM). 

Real time PCR was carried out by incubation of 0.5 μl of cDNA with 8.3 μl of H_2_O, 1.2 μl primers and 10 μl SyBr master mix in a total volume of 20 μl per reaction. mRNA-specific primers for CYP1A1 and CYP1B1 were designed using Primer Express software 2.0. Their sequences are shown in Figure S1. The samples were subjected to 40 cycles of amplification with denaturation at 95 °C for 1 min, annealing temperature at 60 °C for 30 sec and chain elongation at 72 °C for 30 sec. Quantification was achieved by the use of a standard curve for each gene and normalization of CYP1 transcripts by the ΔΔCt method and the use of the housekeeping gene GAPDH. 

### Microsome preparation

Microsomal pellets were prepared as described previously [23]. Tissue samples were weighed, cut into small cubes (1-2 mm) and divided into 0.5 g aliquots. To each sample aliquot 2 ml of ice-cold 10 mM PO_4_ containing 20% glycerol, protease inhibitors and 1.15% (w/v) KCl was added and homogenized using a mechanical homogenizer in 30 second bursts (maximum of 4 bursts). The homogenate was centrifuged for 30 minutes at 9,000 g at 4 °C in a high speed centrifuge and the resulting supernatant (S9 fraction) was centrifuged for 60 minutes at 100,000 g at 4 °C in a Beckman 120 TLX ultracentrifuge. The microsomal pellet was washed once with 400 μl of 10 mM Tris-EDTA buffer (pH 7.4) containing 0.25 M sucrose. Following ultracentrifugation (60 minutes at 100,000 g at 4 °C) the microsomal pellet was resuspended in 10 mM PO_4_ containing 20% glycerol and stored at -80 °C until further use. The protein concentration of each microsomal sample was estimated using the Bradford assay and the determination of P450 content of each sample using the reduced carbon-monoxide spectrum difference of the cytochrome P450. 

### CYP1 enzyme assays

CYP1 enzyme assays were performed using diosmetin as substrate, in the presence of NADPH (5 mM), MgCl_2_ (0.5 mM) and phosphate buffer (20 mM pH 7.4). Recombinant CYP1A1 and CYP1B1 enzymes were used at concentrations of 0.5, 1, 1.5 and 3.5 mg/ml respectively. Microsomal protein derived from tissues or cells was used at a final concentration of 1 mg/ml and the samples were standardized so as to possess equal amounts of CYP content. Following incubation of diosmetin with recombinant CYP1 enzymes at 37 °C, samples were taken at 5, 10, 15, 30 and 60 min intervals. Each reaction (100 μl) was terminated by the addition of equal volume of 1% acetic acid in methanol. The samples were centrifuged at 3,500g for 20 minutes at 4°C and the supernatants were analysed by reversed phase HPLC (Perkin Elmer 200, Wellesley, USA) or mass spectrometry (Shimadzu 2010 EV, Milton Keynes, United Kingdom). For CYP1 enzyme inhibition experiments, the assay was carried out with microsomal protein and diosmetin as described above in the presence of human CYP1B1 antibody (Santa Cruz, Heidelberg, Germany) at 1:500 dilution in PBS 1% FBS. 

### HPLC and mass spec analysis

A similar methodology to that described in previous studies was used [24,25]. A Luna 5µ C_18_ 4.6 x 150mm column (Phenomenex, Cheshire, United Kingdom) was used, with a mobile phase flow rate of 1 ml/min, at a temperature of 37°C. The mobile phase consisted of: Solvent A (1% acetonitrile and 0.5% acetic acid in water), and solvent B (4% acetonitrile and 0.5% acetic acid in methanol). The following gradient program was be used: 60% solvent A and 40% solvent B at time 0, 10% solvent A and 90% solvent B after 10 minutes. Final conditions were held for 1 minute before returning to initial solvent conditions. A calibration curve for diosmetin and luteolin was performed at each run, using the following concentrations of compound standard: 10, 8, 6, 4, 2, 1, 0.1 μM. Diosmetin and luteolin were monitored by UV detection at 360 nm.

Mass spectrometric analysis was run at positive/negative ionization (APCI) and 1.5 kV voltage of the detector, while retaining the initial LC parameter setup, in terms of solvents and gradient.

### Statistical analysis

Kolmogorov-Smirnov test was used to examine whether the data follow normal distribution. Significant differences between mean mRNA and activity levels in tumors and normal samples were investigated using paired t test and Wilcoxon ranks test. Mann-U-Whitney test was employed to examine associations between CYP1 expression and tumor invasion. The level of significance was set at the 95% confidence intervals (p< 0.05). Correlations between mRNA and activity levels in tumor samples were evaluated by Pearson’s correlation coefficient R. The analysis was perfomed using SSPS software (version 15.0.1). 

## Results

### Determination of CYP1A1 and CYP1B1 mRNA levels

**Figure 1:**
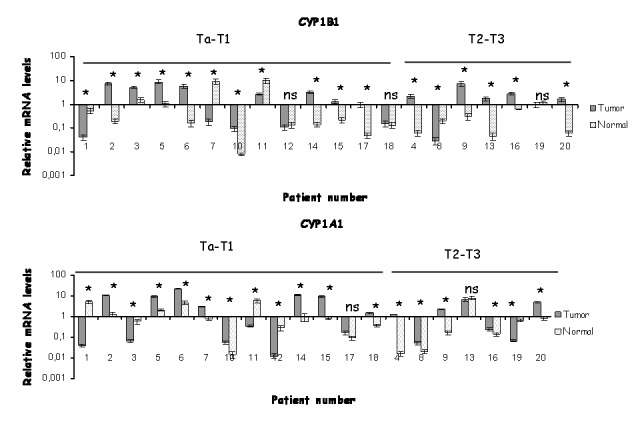
Expression profiling of CYP1A1 and CYP1B1 mRNA in bladder samples. qRT-PCR analysis of CYP1B1 and CYP1A1 in 20 matched normal and tumor pairs derived from bladder tissue. Each bar represents an average of triplicate reactions. The numbers in the X axis correspond to patient numbers. Ta/T1 and T2/T3 represent the different stages of tumors according to TNM classification. ns not statistically significant, * statistically different p< 0.05.

All of 20 matched tumor and normal bladder tissue pairs analyzed expressed detectable levels of CYP1B1 and CYP1A1 mRNA (Figure 1). CYP1A1 and CYP1B1 mRNA showed statistically significant upregulation across the tumor counterpart in 13/20 pairs respectively, as determined by qPCR (Figure 1). This corresponds to an approximate 65% overexpression of CYP1A1 and CYP1B1 mRNA respectively in the tumor tissues. 4/20 and 5/20 tumor tissues revealed significant downregulations of CYP1B1 and CYP1A1 mRNA respectively, whereas in patients 13 and 17 the expression levels of CYP1A1 mRNA between tumor and normal tissue did not reveal a significant change. Similarly the expression of CYP1B1 mRNA in patients 12, 18 and 19 did not exhibit a significant difference between tumor and normal samples. 

**Figure 2:**
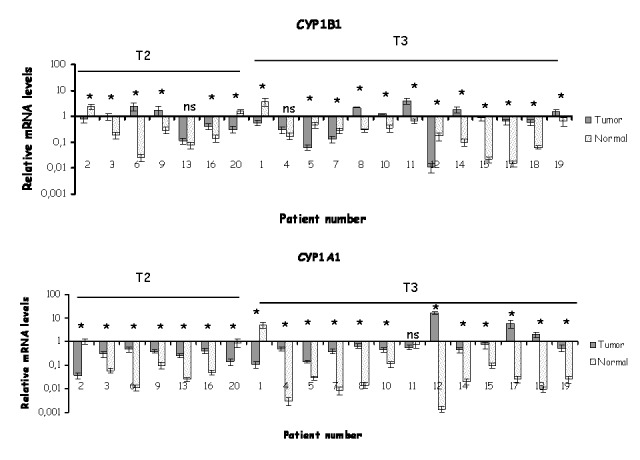
Expression profiling of CYP1A1 and CYP1B1 mRNA in colon samples. qRT-PCR analysis of CYP1B1 and CYP1A1 in 20 matched normal and tumor pairs derived from colon tissue. Each bar represents an average of triplicate reactions. The numbers in the X axis correspond to patient numbers. Ta/T1 and T2/T3 represent the different stages of tumors according to TNM classification. ns not statistically significant, * statistically different p< 0.05.

Colon tumor tissues presented a higher frequency of CYP1A1 overexpression. 16 out 20 (80%) and 12 out of 20 (60%) samples showed significantly higher levels of CYP1A1 and CYP1B1 mRNA respectively in the tumor counterpart (Figure 2). 3 out of 20 samples revealed statistically lower CYP1A1 mRNA levels in tumors compared to normal pairs, while patient number 11 showed no significant change in CYP1A1 mRNA between tumor and normal tissue (Figure 2). CYP1B1 mRNA levels were significantly lower in 6 out of 20 colon tumors compared to normal epithelia, whereas patients 13 and 4 presented non significant difference in CYP1B1 mRNA levels between normal and tumor tissues. 

**Figure 3:**
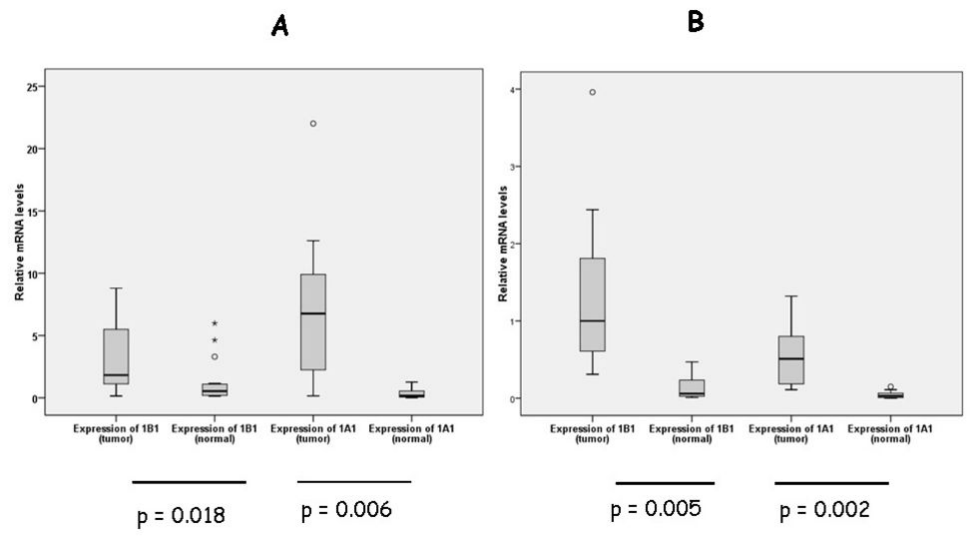
Mean mRNA levels of CYP1A1 and CYP1B1 transcripts in human tumors. Box plots indicate mean ± STDEV for (A) bladder and (B) colon tumor and normal samples. Statistical analysis was conducted using paired T test and Wilcoxon ranks test. Statistical differences were obtained for bladder (n=20) and colon tumors (n=20) vs normals (p< 0.05 for CYP1A1 and CYP1B1).

When mean expression levels of CYP1A1 and CYP1B1 mRNA were compared in the entire panel of bladder or colon tissues the analysis indicated that expression levels of CYP1A1 and CYP1B1 mRNA were higher in bladder and colon tumors compared to normal tissues (p < 0.05, Figure 3A and B).

### Determination of CYP1 activity levels

**Figure 4:**
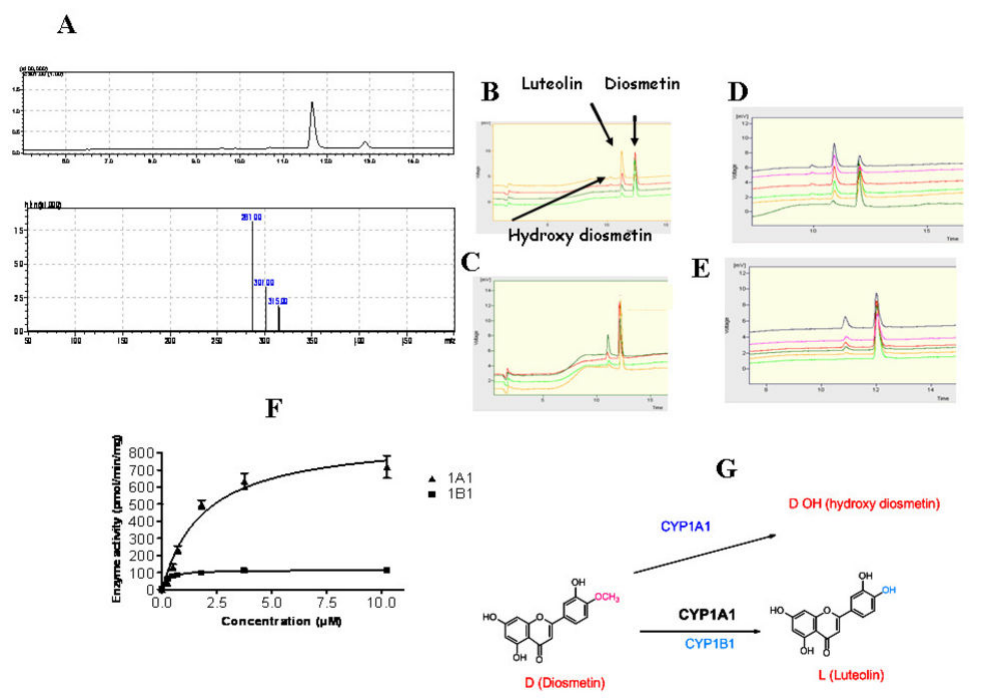
The metabolism of 4´ methoxy 3´,5,7 trihydroxy flavone (diosmetin) by recombinant CYP1A1 and CYP1B1. Diosmetin (10 μM) was incubated with recombinant CYP1A1 (1mg/ml) and CYP1B1 (1mg/ml) at various time points in the presence of NADPH (5mM) and MgCl2 (0.5mM) as described in Materials and Methods. (A) LC-MS analysis of diosmetin metabolism by CYP1A1. Mass spectrometric trace indicated two positively charged ions (287, 301) and one negatively charged ion (315) that correspond to diosmetin, 3´,4´,5,7 tetrahydroxy flavone (luteolin) and hydroxy - 4´ methoxy tetrahydroxy flavone (hydroxy diosmetin) respectively. (B) Enzyme profile of diosmetin metabolism by CYP1A1. Diosmetin was incubated for 20 min with increasing enzyme concentrations corresponding to 0.5, 0.8, 1.2 and 3,5 mg/ml. Green line to orange line: increasing concentrations of CYP1A1 recombinant enzyme. (C) Enzyme profile of diosmetin metabolism by CYP1B1. Diosmetin was incubated for 20 min with increasing enzyme concentrations corresponding to 0.5, 0.8, 1.2 and 3,5 mg/ml. Orange line to black line: increasing concentrations of CYP1B1 recombinant enzyme. (D) Time dependence analysis of diosmetin (10 μM) metabolism by CYP1A1 at 1, 5, 10, 15, 30, 60 min. Black to blue line: 1-60 min. (E) Time dependence analysis of diosmetin metabolism by CYP1B1 (1mg/ml) at 1, 5, 10, 15, 30, 60 min time intervals. Black to blue line: 1-60 min. (F) Michaelis-Menten kinetics of luteolin production by CYP1A1 and CYP1B1-mediated metabolism of 4´ methoxy 3´,5,7 trihydroxy flavone indicating maximum activity (Vmax).

**Figure 5:**
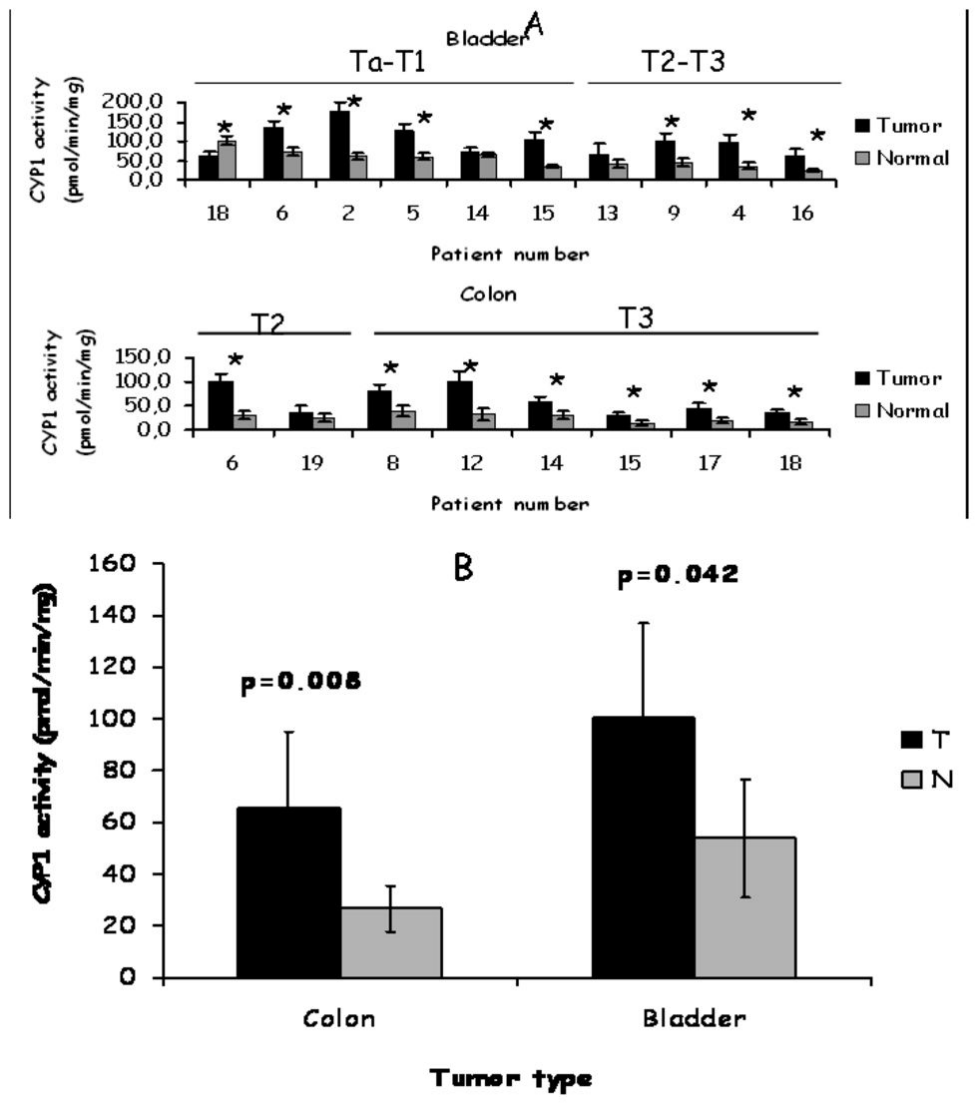
Determination of CYP1 expression by enzyme activity assay. The X axis corresponds to patient numbers while the Y axis to CYP1 activity levels. Activity was calculated from production of the metabolite luteolin per time per amount of microsomal protein. Each bar represents an average of triplicate reactions. Ta-T1 and T2-T3 correspond to different stages of tumors according to TNM. (A) Bladder samples (n=10). Colon samples (n=8). ns not statistically significant, * statistically different p< 0.05. (B) Mean CYP1 activity levels in human tumors. Bar charts indicate mean ± STDEV for bladder (n=10) and colon (n=8) tumors and normal samples. Statistical analysis was conducted using paired T test and Wilcoxon ranks test. Statistical differences were obtained for bladder and colon tumors vs normals (p<0.05).

The expression of CYP1 enzymes was determined by an activity assay that was based on the demethylation of diosmetin [24]. Metabolism of the substrate by CYP1A1 and CYP1B1 yields the product luteolin, albeit to different extents (Figure 4). Metabolism of diosmetin by CYP1A1 yields hydroxy diosmetin to lesser amounts, in addition to luteolin. The production of luteolin and hydroxy diosmetin was verified by mass spectrometry (Figure 4). All parameters required for optimum enzyme activity measurements were optimized in preliminary experiments (Figure 4). CYP1 activity in tumors was estimated by the amount of product formed per time per protein concentration (Figure S2). In contrast to qRT-PCR results, 10 out of 20 bladder samples did not express active CYP1 enzymes, to sufficient levels that could be detected by the assay used (data not shown). Activity levels were significantly higher in 7 out of 20 (35%) and lower in 1 out of 20 bladder tumors (Figure 5A). 2 out 20 bladder samples did not show a significant change between tumor and normal part (Figure 5A). Similarly 7 out of 20 (35%) colon tumors exhibited higher CYP1 activity compared to their corresponding controls, whereas patient 19 revealed no significant difference in activity between normal and tumor part (Figure 5A). No detectable CYP1 activity was found in 12 out of 20 colon samples (data not shown, for clarity). Mean CYP1 activity of bladder and colon tumor samples was statistically different compared to CYP1 activity found in the corresponding healthy tissues (p < 0.05) (Figure 5B). 

In addition to diosmetin, incubations of tumor microsomes were performed in the presence of 7-ethoxyresorufin. 7-ethoxyresorufin is a model substrate for CYP1A1 and CYP1B1 enzymes, as reported by Waxman and colleagues [26]. Although EROD activity levels were somewhat higher compared to CYP1 activity estimated by diosmetin demethylation the overall overexpression difference between normal and tumor part remained similar to that noted before (Figure S3). 

### Relationship between CYP1 enzyme expression and tumor pathology

**Figure 6:**
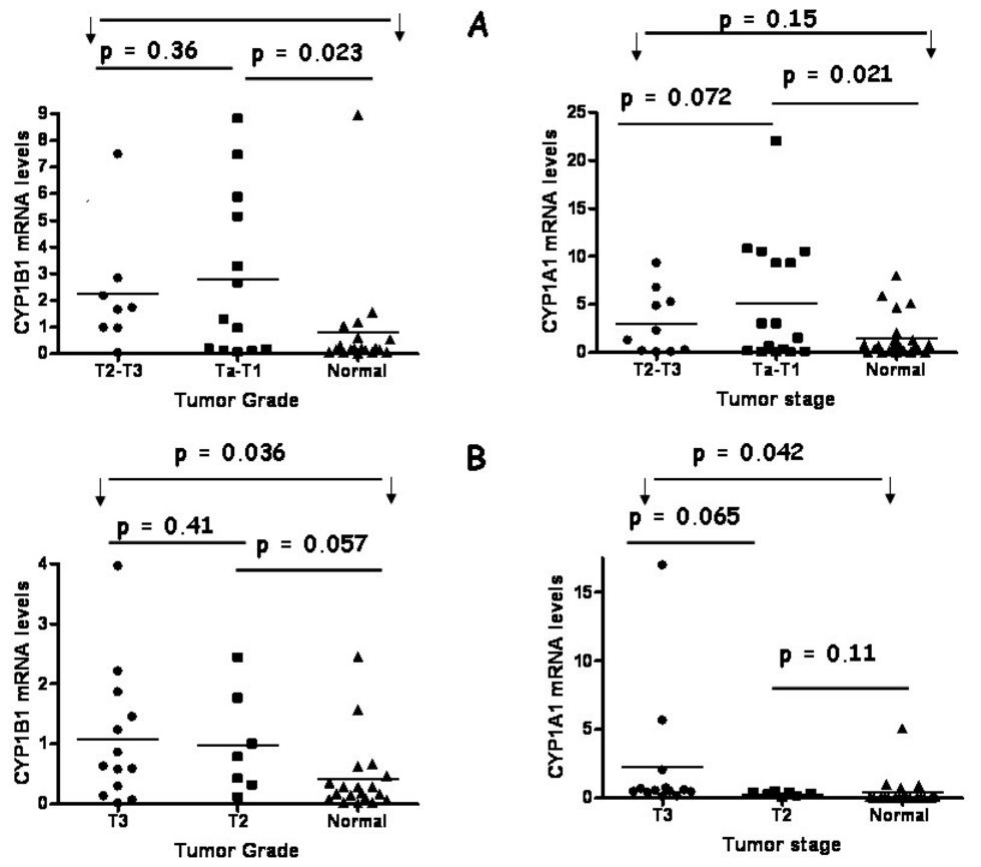
Correlation of CYP1 mRNA expression levels with tumor stage. Group pairs were compared using Mann-U-Whitney test. Bars depict the mean values. Scatterplot depicting mRNA levels of CYP1A1 and CYP1B1 genes in tumor samples of different TNM status and normal samples of (A) bladder and (B) colorectal origin. Statistical significance was set at p < 0.05. Arrows and horizontal lines indicate groups compared with statistical tests.

The mRNA expression of the CYP1 genes was further investigated relative to the stage of the tumors. Tumor stage was grouped to Ta-T1 and T2-T3 in bladder tumors, due to low number of Ta and T2 samples. Statistically significant differences in expression of each gene among the tumor groups Ta-T1/T2-T3 in bladder and T2/T3 in colon are depicted in Figure 5. CYP1B1 and CYP1A1 mean mRNA levels in bladder tumors were not associated with tumor invasion, as non significant differences were obtained between T2-T3 tumors and Ta-T1 tumors (p= 0.36 and p= 0.072 respectively) (Figure 6A). Significant differences were obtained only between Ta-T1 and normal groups, with regard to CYP1B1 and CYP1A1 mean mRNA levels (p =0.023 and p = 0.021 respectively, Figure 6A). A similar result was obtained for colon tumors. Mean CYP1B1 and CYP1A1 mRNA levels of T3 and T2 colon tumors did not show a significant difference (p =0.41 and p= 0.065 respectively, Figure 6B). Significant differences were obtained between T3 and normal groups with regard to mean CYP1A1 (p =0.042) and mean CYP1B1 (p=0.036) mRNA levels (Figure 6B). Overall the data demonstrated that CYP1 mRNA levels were not associated with tumor invasion in colon or bladder samples. 

**Figure 7:**
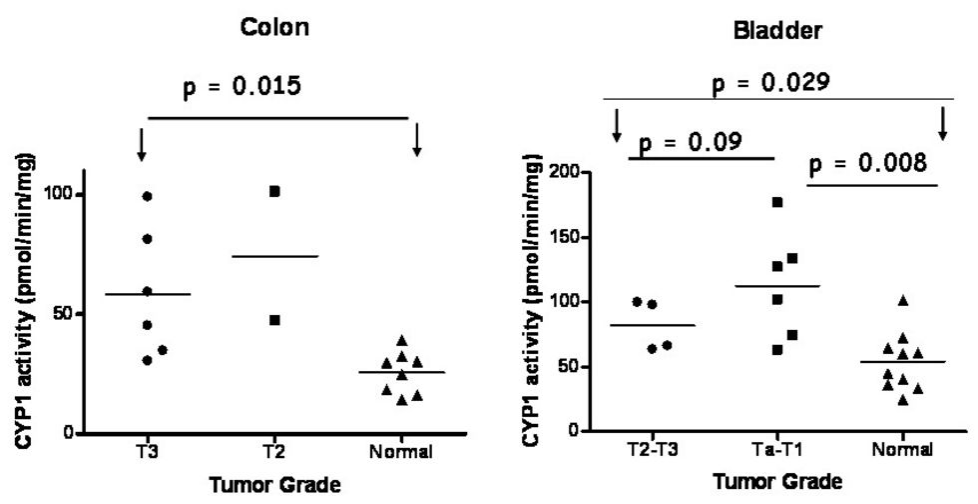
Correlation of CYP1 enzyme activity levels with tumor stage. Group pairs were compared using Mann-U-Whitney test. Bars depict the mean values. Scatterplot depicting CYP1 activity levels in tumor samples of different TNM status and normal samples of bladder and colorectal origin. Statistical significance was set at p < 0.05. Arrows and horizontal lines indicate groups compared with statistical tests.

In addition to CYP1 mRNA, CYP1 activity was investigated relative to the stage of the tumors. Fewer samples were acquired for statistical analysis, since not all tissues expressed active CYP1 enzymes. Ta-T1 bladder tumors possessed significantly higher levels of CYP1 enzyme activity compared to the control group (p=0.008) (Figure 7). When T1-Ta and T2-T3 groups were compared no significant differences were obtained (p=0.09) (Figure 7). With regard to colon tissues no significant relationship was obtained for T3 (n=5) tumors and control normal samples (p= 0.015) whereas the T2-group was composed of only 2 samples, thus no statistical analysis was possible (Figure 7).

### CYP1 activity in human tumors is mainly indicative of active CYP1B1

**Figure 8:**
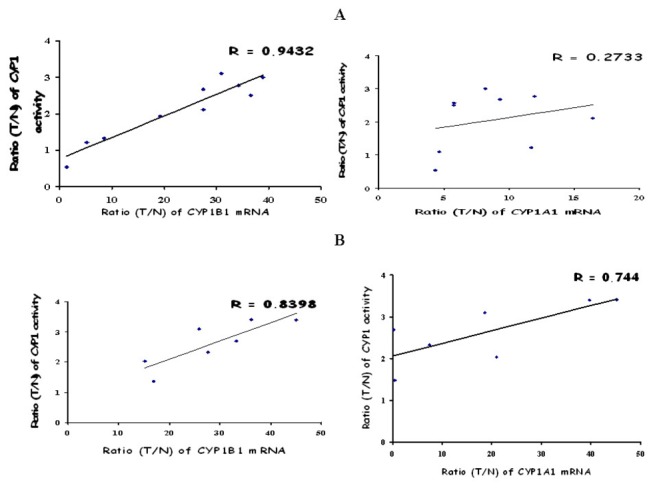
CYP1 activity is mainly indicative of active CYP1B1 in human tumors. Correlation of CYP1A1 and CYP1B1 mRNA T/N expression ratio with CYP1 activity T/N expression ratio in (A) bladder and (B) colon tumors using linear regression analysis.

**Figure 9:**
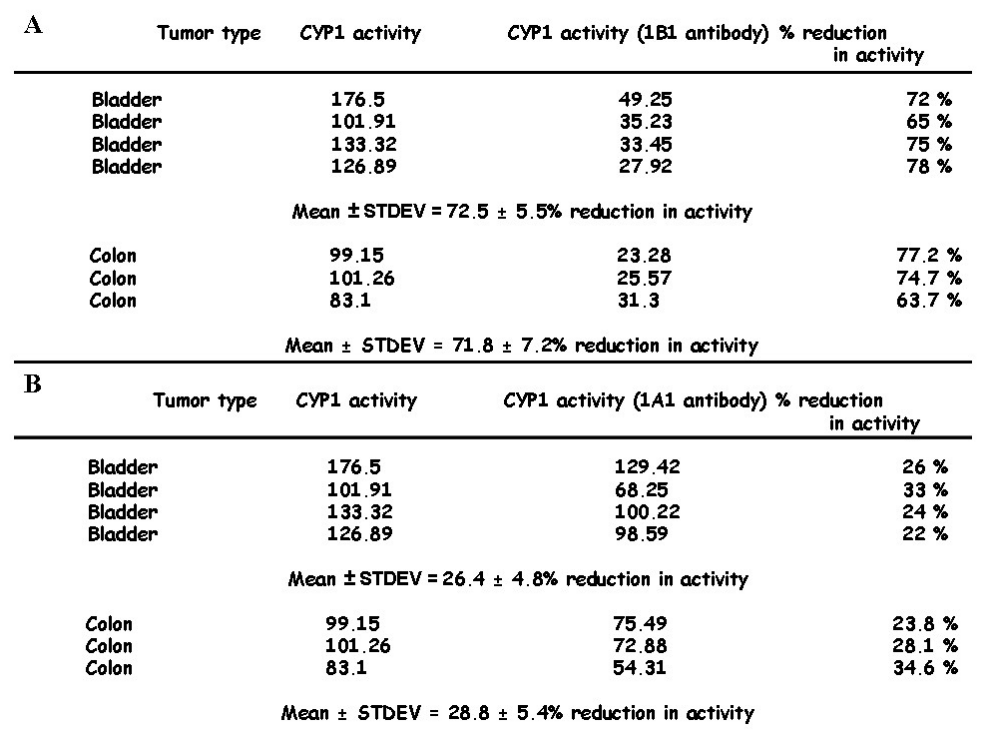
Reduction of CYP1 activity in colon (n=3) and bladder tumors (n=4) by (A) CYP1B1 antibody (1:500) (B) CYP1A1 antibody (1:300).

It is important at this point to state that no CYP1 enzymatic assay is reported that differentiates between CYP1A1 and CYP1B1 enzyme activity. Based on the primary analysis of CYP1-mediated metabolism of diosmetin in human tumors (Figure S2) we hypothesized that active CYP1B1 is expressed at higher levels in tumor samples. In order to substantiate this hypothesis linear regression analysis between active CYP1 enzyme expression levels of tumors and their corresponding CYP1A1 and CYP1B1 mRNA levels was performed. Expression levels were presented as the ratio between Tumor/Normal expression of mRNA or enzymatic activity. Pearson correlation coefficients between CYP1B1 mRNA T/N expression ratio and T/N expression ratio in CYP1 activity of bladder tumors were higher than those corresponding to CYP1A1 mRNA T/N expression ratio levels (R = 0.94 vs 0.27 Figure 8A). Colon tumors revealed a higher correlation coefficient between T/N expression ratio of CYP1A1 mRNA and T/N expression ratio of CYP1 activity compared to bladder tumors (R = 0.74, Figure 8B). However the latter value was lower than the coefficient noted in the case of CYP1B1 (R = 0.83, Figure 8B). To further prove that CYP1B1 is notably responsible for the activity determined in tumors, we performed incubation of microsomes isolated from cancer tissue with the substrate diosmetin, in the presence of a polyclonal CYP1B1 antibody (Santa Cruz, Heidelberg, Germany) at 1:500 dilution. Co-incubation of CYP1B1 antibody with tumor microsomal protein of 4 bladder and 3 colon samples expressing high levels of CYP1 enzyme activity resulted in a 72.5 ± 5.5 % (bladder) and 71.8 ± 7.2 % (colon) reduction in catalytic activity indicating that the activity levels were attributed mainly to CYP1B1 expression (Figure 9A and S4). 

To further substantiate that CYP1A1 is a minor contributor to CYP1 activity noted in tumors we performed incubations of tumor microsomes with diosmetin in the presence of a polyclonal antibody raised against human CYP1A1 purchased from Millipore (AB 1258, Massachusetts, US). Our analysis showed that co-incubation of CYP1A1 antibody reduced the activity albeit to levels that were lower to those observed in the case of the CYP1B1 antibody (Figure 9B). Overall CYP1 activity was reduced to 26.3 ± 4.8% in n=4 bladder tumors and 28.8 ± 5.4% in n=3 colon tumors (Figure 9B). 

**Figure 10:**
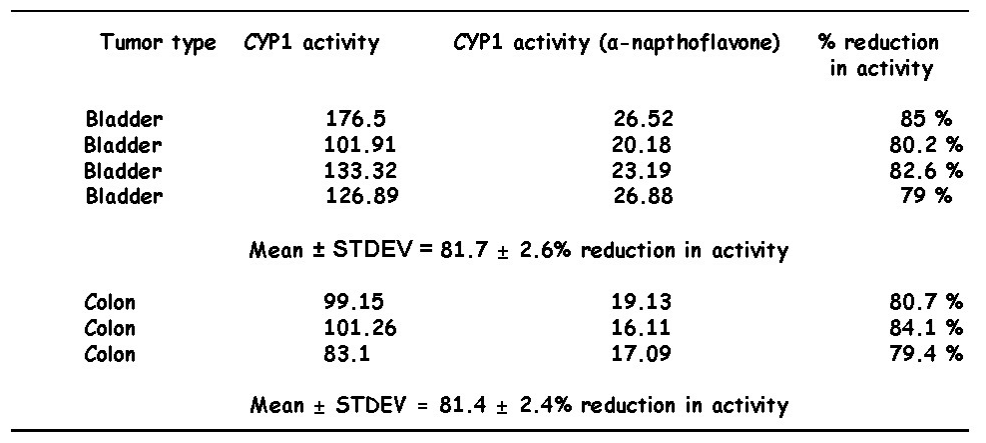
Reduction of CYP1 activity in colon (n=3) and bladder tumors (n=4) by α-napthoflavone (0.5 μM).

Incubations of tumor microsomes with diosmetin in the presence of the CYP1 inhibitor α-napthoflavone resulted in stronger inhibition of the formation of luteolin. The inhibition was higher than that noted in the case of the CYP1B1 antibody. Overall inhibition of activity in tumors expressing high CYP1 activity was reduced to 81.7 ± 2.6% in n = 4 bladder tumors and to 81.4 ± 2.4% in n = 3 colon tumors (Figure 10). 

## Discussion

In the current study an expression analysis of CYP1 enzymes in human tumors of bladder and colorectal origin was undertaken. CYP1A1 and CYP1B1 were differentially overexpressed in colon and bladder tumors. More importantly the presence of active CYP1 enzyme expression was detected at significantly higher levels in bladder and colon tumors compared to normal epithelia. CYP1 activity was mainly attributed to CYP1B1 expression. Several recent and early reports have focused on the expression pattern of CYP1B1 in human tumors. Differential overexpression of CYP1B1 has been demonstrated, in terms of mRNA and protein levels in a variety of human tumors of different origin by Murray and colleagues [12-14,27-29]. As a result CYP1B1 is considered a potential tumor marker and a putative target for cancer therapy. 

The results of our analysis corroborate with these findings, given that CYP1A1 and CYP1B1 mRNA transcripts were elevated in 80% and 60% of colon and in 65% of bladder tumors respectively. Lower percentages of tumor samples (35% for colon and 35% for bladder respectively) exhibited differentially expressed CYP1 activity. Our observations further demonstrate that not all CYP1 mRNA is translated to fully active enzyme. A similar conclusion has been deduced by the studies of Sissung et al. and Spink et al., where it was shown that CYP1B1 mRNA detection does not always correlate with CYP1B1 protein expression [15,30]. In addition several studies have produced conflicting results regarding the expression of CYP1B1 to the mRNA and protein level [12,14,27,31]. In our analysis we observed differential mRNA overexpression that did not always correlate with activity overexpression. A possible reason that will explain such observations could be attributed to the basal levels of CYP1B1 mRNA that require a certain threshold before the protein can be expressed, as suggested by Sissung et al. [30]. A second explanation may include cell-specific post-translational modifications before protein expression is achieved as well as proteolytic degradation that modulates the enzyme’s protein levels [32,33]. In addition, polymorphisms in CYP1 genes are important determinants of CYP1 enzyme activity and consequently CYP1-catalyzed metabolism of xenobiotics. It is well established that some polymorphic variants of the CYP1A1 enzyme such as the CYP1A1.4 Thr461Asn show reduced catalytic activity towards CYP1A1 substrates such as oestradiol [34]. Similarly the variants CYP1B1.6 and CYP1B1.7 show reduced Vmax and increased Km, i.e. reduced clearance and consequently reduced catalytic activity towards the substrate oestradiol, compared to the wild type CYP1B1.1 [35]. In this context it is possible that such variants may be incapable of metabolizing CYP1 substrates such as diosmetin effectively thus resulting in reduced formation of the metabolite luteolin and consequently reduced CYP1 activity. Similar to our study, increased expression of CYP1B1 in colon tumors has been characterized by Kumarakulasingham et al. using immunohistochemical staining techniques in 264 subjects with primary colorectal cancer [36]. Moreover Gibson and colleagues demonstrated strong CYP1B1 immunoreactivity present in human colorectal tumor epithelia of 61 subjects and absence of CYP1B1 staining in normal colonic epithelia [37]. In contrast, downregulation of CYP1B1 expression in oral squamus cell carcinoma and endometrial cancer was documented by Pradhan et al. and Hevir et al. respectively [38,39]. The exact aetiology for such contradictory observations is not clear. It has been suggested that CYP1B1 levels are influenced by exogenous stimulation of the AhR-xenobiotic-pathway by polycyclic aromatic hydrocarbons and related environmental contaminants [38,40]. Consequently CYP1B1 expression in patients with cancer varies amongst other factors, according to lifestyle and social habits. Apart from the activation of the AhR pathway, epigenetic silencing occurring due to promoter methylation of CYP1B1 in tumors can account for putative downregulation of CYP1B1 [41]. 

The most significant finding of the current study is the evidence regarding the activity levels of CYP1 enzymes in human tumors. This is one of the few studies that substantiate the implication of CYP1B1 and CYP1A1 to a lesser extent, in cancer therapy due to their differential enzyme activity overexpression in bladder, and colon tumors. Although overexpression of CYP1B1 and CYP1A1 in tumors has been demonstrated at the mRNA and protein level [12,14,28,42] information regarding enzymatic activity is scarce. Detection of CYP1B1 or CYP1A1 protein with the use of CYP-specific antibodies, does not guarantee that expression levels are translated fully to active enzyme. To our knowledge there is no study to date that has provided evidence regarding the activity levels of CYP1B1 and CYP1A1 enzymes in bladder and colon tumors. Our data support the use of CYP1 and mainly CYP1B1 enzymes in cancer therapy, via the selective activation of non cytotoxic prodrugs to toxic metabolites. Such strategy has been employed by the studies of Sheldrake et al., Travica et al., Pors et al., Meng et al., Sutherland et al. and Androutsopoulos et al., for the development of novel selective CYP1-targeted cancer therapeutics [19-21,43-45]. Specifically the studies by Sheldrake et al., Travica et al., Pors et al. and Sutherland et al. have focused on the development and optimization of novel duocarmycin analogues that are activated by CYP1A1 or CYP2W1 into potent cytotoxins, as new anticancer prodrugs for bladder or colon cancer treatment. 

Two early reports from the group of Liehr and Ricci demonstrated overexpression of CYP1B1 activity in mammary adenocarcinoma and human uterine myoma, compared to normal breast tissue and surrounding myometrial tissue respectively [23,46]. The activity assay used in the latter two studies was based on the 4-hydroxylation of oestradiol by CYP1B1, however the metabolites were detected by Thin Layer chromatography, that is much less sensitive and informative than High Pressure liquid chromatography. A recent study performed by the group of Murray identified CYP1B1 activity in renal cell carcinoma using an activity assay based on the O-deethylation of 7-ethoxyresorufin and the use of the CYP1A1/CYP1B1 inhibitor α-napthoflavone [47]. The results presented herein highlight an additional activity assay based on the demethylation of diosmetin. Activity of CYP1 enzymes can be detected by the production of the metabolite of luteolin and quantification via calibration curves containing authentic standards of both compounds. Incubation of tumor microsomal protein with diosmetin and CYP1B1/CYP1A1 specific antibodies can improve the selectivity of the assay towards CYP1A1 and CYP1B1 isoforms.

Statistical analysis performed in the current study revealed that CYP1A1 and CYP1B1 mRNA expression is not associated with tumor invasion in bladder and colon tumors, as indicated by Figure 6 where TNM status is compared to CYP1 expression levels. In addition comparison of CYP1 activity levels with tumor stage did not produce significant differences. Lack of association between CYP1B1 protein expression and stage 1 or 2 oral squamus cell carcinoma tumors was demonstrated in the study by Pradhan and colleagues in a sample size of 36 cases and their corresponding controls, suggesting that CYP1B1 expression is independent of TNM status and site of lesion [38]. Gibson and coworkers reported in 2003 that CYP1B1 protein expression does not possess a direct role in tumor invasion as the level of CYP1B1 expression in stage 1 (pT1 and pT2) was not significantly different than that of stage 2 colon tumors (pT3 and pT4) in a sample size of 61 colon tumors [37]. These observations corroborate well with the findings of the current study with respect to tumor invasion and CYP1 expression. In contrast Kumarakulasingham et al. reported significant correlations for CYP1B1 but not for CYP1A1 between the expression of this protein in the primary tumor and the expression in the secondary metastatic tumor in 264 subjects with primary colon cancer and 91 subjects with lymph node metastasis [36]. Thus a major factor that determines the outcome of the statistical analysis is the total sample size of tumor specimens and consequently the sample size corresponding to each tumor stage (T1, T2, T3 and T4). The current study and the study performed by Pradhan and colleagues suffer from the disadvantage that the sample size of each tumor stage is too low. Based on these data it is uncertain whether CYP1 enzyme expression can influence the invasion and severity of bladder and colon tumors and further studies are required to prove this hypothesis. 

In conclusion the present study provides information on the expression pattern of the two extrahepatic members of the CYP1 family in human tumors of different origin. The finding that CYP1B1 active protein is differentially expressed in bladder and colon tumors strongly supports its use for anticancer therapy. The data adds interesting insight into the contribution of CYP1 expression in cancer pathology and the potential use of CYP1 and mainly CYP1B1 enzymes in cancer therapy. 

## Supporting Information

Figure S1 **qPCR assay for CYP1A1 and CYP1B1 mRNA detection in tumor samples.** Amplification plots and dissociation curves derived from 3 samples isolated from tumors indicating the formation of a single product corresponding to CYP1A1 and CYP1B1 mRNA detection. PCR was conducted at 60 °C annealing temperature for each primer set. The size of the products was confirmed by gel electrophoresis.
(JPG)

Figure S2 **CYP1 activity detection in human tumors.** Metabolism of diosmetin to the metabolite luteolin by bladder tumor and normal microsomes. Top trace indicates a bladder sample of “high” CYP1 activity (176 pmol/min/mg), whereas bottom trace indicates a bladder sample of “low” CYP1 activity (34 pmol/min/mg).
(JPG)

Figure S3 **Determination of CYP1 expression by EROD enzyme activity assay.** The X axis corresponds to patient numbers while the Y axis to CYP1 activity levels. Activity was calculated from production of the metabolite resorufin per time per amount of microsomal protein.
(JPG)

Figure S4 **CYP1 activity is mainly indicative of active CYP1B1 in human tumors.** HPLC trace depicting the metabolism of diosmetin to luteolin in microsomes isolated from a colon tumor of high CYP1 activity in the presence and absence of CYP1B1 (1:500) polyclonal antibody (Santa Cruz, Heidelberg, Germany).
(JPG)
