# Supplementary figures and images for: Expression Profile of CYP1A1 and CYP1B1 Enzymes in Colon and Bladder Tumors
Source: PLoS One. 2013 Dec 16;8(12):e82487. doi: 10.1371/journal.pone.0082487 (PMC3864999; doi:10.1371/journal.pone.0082487)

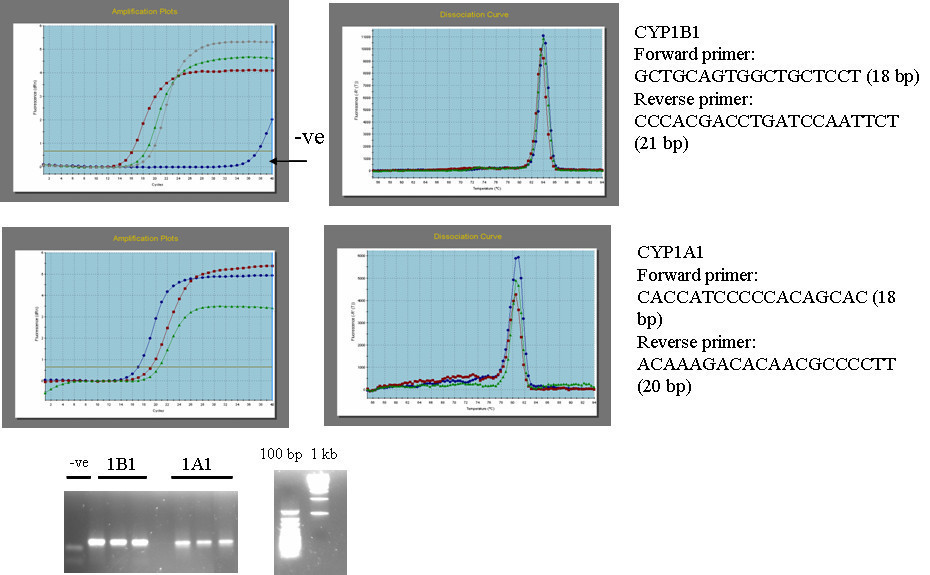

Supplement: Figure S1 — qPCR assay for CYP1A1 and CYP1B1 mRNA detection in tumor samples. Amplification plots and dissociation curves derived from 3 samples isolated from tumors indicating the formation of a single product corresponding to CYP1A1 and CYP1B1 mRNA detection. PCR was conducted at 60 °C annealing temperature for each primer set. The size of the products was confirmed by gel electrophoresis. (JPG) [file pone.0082487.s001.jpg]

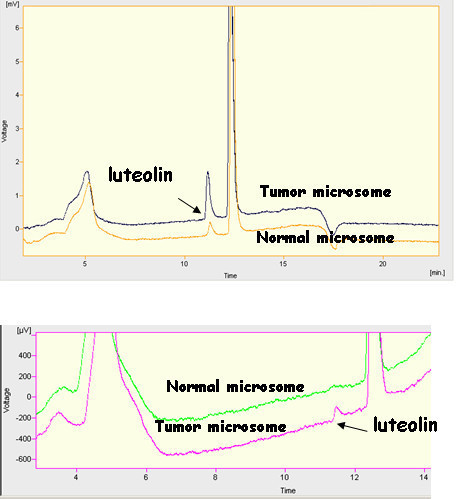

Supplement: Figure S2 — CYP1 activity detection in human tumors. Metabolism of diosmetin to the metabolite luteolin by bladder tumor and normal microsomes. Top trace indicates a bladder sample of “high” CYP1 activity (176 pmol/min/mg), whereas bottom trace indicates a bladder sample of “low” CYP1 activity (34 pmol/min/mg). (JPG) [file pone.0082487.s002.jpg]

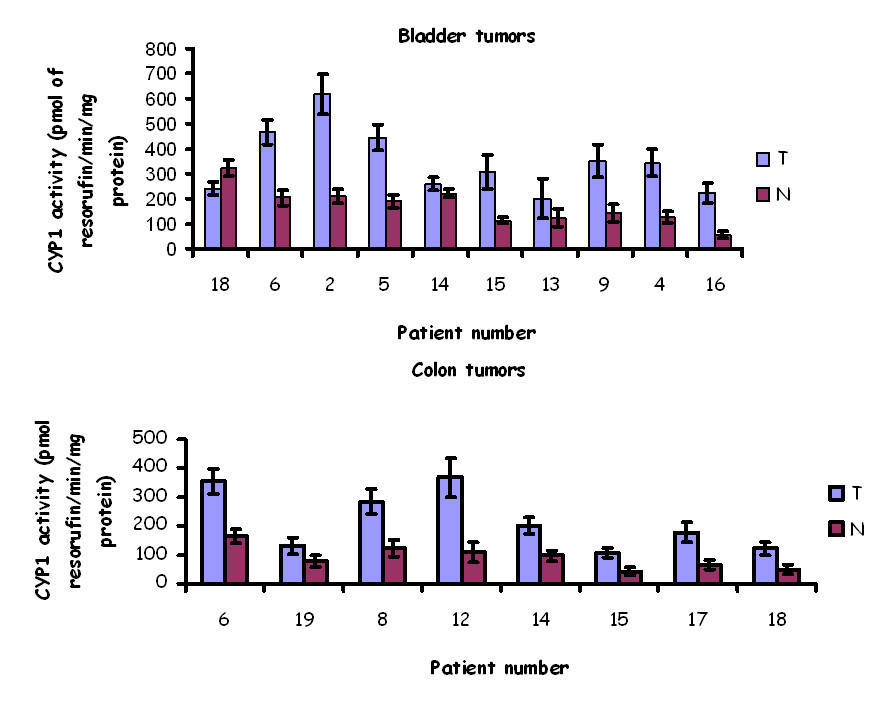

Supplement: Figure S3 — Determination of CYP1 expression by EROD enzyme activity assay. The X axis corresponds to patient numbers while the Y axis to CYP1 activity levels. Activity was calculated from production of the metabolite resorufin per time per amount of microsomal protein. (JPG) [file pone.0082487.s003.jpg]

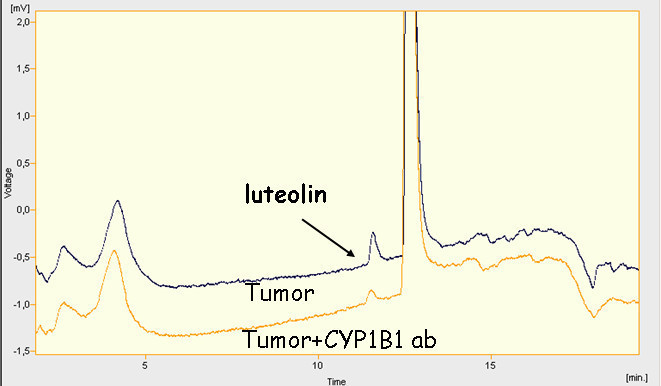

Supplement: Figure S4 — CYP1 activity is mainly indicative of active CYP1B1 in human tumors. HPLC trace depicting the metabolism of diosmetin to luteolin in microsomes isolated from a colon tumor of high CYP1 activity in the presence and absence of CYP1B1 (1:500) polyclonal antibody (Santa Cruz, Heidelberg, Germany). (JPG) [file pone.0082487.s004.jpg]
